# Analysis of the myeloid-derived suppressor cells and annexin A1 in multibacillary leprosy and reactional episodes

**DOI:** 10.1186/s12879-021-06744-x

**Published:** 2021-10-09

**Authors:** Stephanni Figueiredo da Silva, Leticia Rossetto da Silva Cavalcante, Ezequiel Angelo Fonseca Junior, Joselina Maria da Silva, José Cabral Lopes, Amilcar Sabino Damazo

**Affiliations:** 1 Post-Graduate Program in Health Sciences, Faculty of Medicine, Universidade Federal de Mato Grosso (UFMT), 2367 Fernando Correa da Costa Avenue, Cuiabá, MT 78060-900 Brazil; 2 Coordenator of the Laboratory of Clinical Analysis in Jangada, Jangada, MT 78490-000 Brazil; 3 Ambulatory of Leprosy, University Hospital Julio Müller, Luis Philippe Pereira Leite Street, Cuiabá, MT 78048-902 Brazil; 4 Post-Graduate Program in Professional Master in Science Applied at Hospital Attention, University Hospital Julio Müller, Luis Philippe Pereira Leite Street, Cuiabá, MT 78048-902 Brazil; 5 Department of Basic Sciences in Health, Faculty of Medicine (FM), School of Medicine, Universidade Federal de Mato Grosso (UFMT), 2367 Fernando Correa da Costa Avenue, MT 78060-900 Cuiabá, Brazil

**Keywords:** *Mycobacterium leprae*, lepromatous leprosy, Myeloid-derived suppressor cells, Annexin A1

## Abstract

**Background:**

Leprosy is a chronic infectious disease caused by *Mycobacterium leprae*. Patients have distinct clinical forms, and the host´s immunological response regulate those manifestations. In this work, the presence of the myeloid-derived suppressor cell and the regulatory protein annexin A1 is described in patients with multibacillary leprosy and with type 1 and 2 reactions.

**Methods:**

Patients were submitted to skin biopsy for histopathological analysis to obtain a bacilloscopic index. Immunofluorescence was used to detect myeloid-derived suppressor cells and annexin A1.

**Results:**

The data demonstrated that the presence of granulocytic and monocytic myeloid-derived suppressor cells in leprosy patients. A high number of monocytic myeloid-derived suppressor cells were observed in lepromatous leprosy and type 2 reactional patients. The presence of annexin A1 was observed in all myeloid-derived suppressor cells. In particular, the monocytic myeloid-derived suppressor cell in the lepromatous patients has higher levels of this protein when compared to the reactional patients. This data suggest that the higher expression of this protein may be related to regulatory response against a severe infection, contributing to anergic response. In type 1 reactional patients, the expression of annexin A1 was reduced.

**Conclusions:**

Myeloid-derived suppressor cell are present in leprosy patients and annexin A1 might be regulated the host response against *Mycobacterium leprae*.

**Supplementary Information:**

The online version contains supplementary material available at 10.1186/s12879-021-06744-x.

## Introduction

Leprosy is a chronic infectious disease caused by *Mycobacterium leprae*. Transmission occurs by very close and prolonged coexistence with non-treated pauci or multibacillary leprosy patients [1, 2]. Patients have distinct clinical forms, affecting mainly the skin and the nerves, causing lesions resulting from inflammatory processes [3, 4]. The extension of lesion may be related to the genetic background and immune response of the host [4, 5]. Some individuals, in the beginning, during or after the end of the treatment, may present acute clinical manifestations due to the release of antigens and hypersensitivity reactions, known as leprosy reactions: type 1 reaction (T1R) or reversal reaction, and type 2 reaction (T2R) or erythema nodosum leprosum (ENL) [6, 7].

Earlier studies have shown that immunoregulatory cells called myeloid-derived suppressor cells (MDSCs) are heterogeneous population of immature cells that exist as two main subtypes, the granulocytes (G-MDSC), and monocytes (M-MDSC), with potent immunosuppressant activity and may influence the outcome of infectious diseases [7, 8]. MDSC activation was involved in conditions such as transplants, cancer, and some acute and chronic infections [9–12]. Recently, MDSCs have been shown to be essential cells in counter-balancing inflammatory responses and pathogenesis during infections [13].

MDSCs release high levels of cytokines such as interleukin (IL)-10, interferon (IFN)-γ and transforming growth factor (TGF)-β [10, 14–16]. Also, they inhibit T lymphocyte activity through various mechanisms. For example, M-MDSCs produce nitric oxide (NO) via inducible NO synthase (iNOS), whereas G-MDSCs produce reactive oxygen species (ROS), express arginine (Arg)-1 and reduce the levels of L-arginine [10, 16, 17].

In this context, annexin-A1 (ANXA1), a leukocyte regulatory and anti-inflammatory protein have been studied [18, 19]. Some works have described that ANXA1 expression of Treg cells could enhance its inhibitory function of cytokines [20, 21]. Other work with transgenic mice deficient in ANXA1 indicates that T cell increased its effects on intracellular signalling, proliferation, and Th1/Th17 cytokine release [22]. However, no previous work has described the presence of ANXA1 in MDSC.

Thus, this work aimed to identify the presence of MDSCs and the protein ANXA1 in leprosy patients with clinical forms of leprosy and with T1R and T2R.

## Methods

### Patients

This was a prospective study. Eligible leprosy patients were diagnosed with tuberculoid (TT), borderline (BB), lepromatous (LL) and with T1R and T2R (n = 170) in the years between 2017 and 2019 in the clinic of infectious diseases at the University Hospital Júlio Müller (UHJM) in Cuiabá, MT, Brazil. The patients were diagnosed according to the criteria established by Ridley and Jopling [4].

At the time of collection, the TT, BB and, LL patients were naïve to treatment. Since a considerable number of patients who develop a leprosy reaction do so after starting treatment, those with T1R and T2R had already started multidrug therapy for leprosy and were also receiving treatment for the reaction episode with corticosteroids or thalidomide, respectively.

All T1R were clinically diagnosed as borderline-borderline. In T2R, 5 patients were borderline lepromatous, and 20 were lepromatous leprosy patients.

Individuals between 18 and 70 years of age were included in this study. Pregnant or lactating women and patients co-infected with seropositivity for human immunodeficiency virus (HIV) or other parasitic diseases were excluded.

For data collection, a standard questionnaire was used with the following information: age, skin color, sex, characteristics of the lesion and region of the affected nerves [23].

All patients were submitted to general physical and dermato-neurological examination by the physician responsible for the service. General health conditions, characterization of the lesion (location, size, edges, and thermal, painful and tactile sensitivity), evaluation of nerve thickening, and sensitivity tests in members through the Semmes–Weinstein monofilaments were evaluated [24]. Participants who agreed to participate in the study signed the informed consent form, approved by the Committee for Ethics in Research of UHJM (CAAE No. 45051415.5.0000.5541), taking into account Resolutions no. 466/12 of the Brazilian Health Council and international ethical guidelines (Declaration of Helsinki).

### Collection of biological material

Tissue samples were collected at the time of diagnosis of leprosy. The procedure was initiated by the asepsis and local anesthesia with 2% lidocaine without vasoconstrictor, performing a biopsy using a “punch” of 4 mm at the edge of the lesion with a sign of clinical activity. The tissue fragment was immersed in 4% buffered (phosphate buffer saline, PBS) paraformaldehyde and transported to the Laboratory of Histology of the Faculty of Medicine, Federal University of Mato Grosso (UFMT), Cuiabá, Brazil, for diagnosis.

### Histological analysis

The samples were washed in the same buffer, dehydrated in solutions with increasing ethanol concentration, cleared in xylene, and embedded in paraffin. Paraffin sections were obtained in the microtome HIRAX M60 (Carl Zeiss, Germany), placed on histological slides, rehydrated, and stained with hematoxylin–eosin for histopathological analysis. Another section was stained with Fite-Faraco, for acid-alcohol-fast bacilli (AFB) analysis. Morphological and quantification of the bacillary index were done under a microscope. The results were expressed on a logarithmic scale of Ridley and Jopling [4].

### Quantification of endogenous ANXA1 expression, and identification of M-MDSC and G-MDSC by immunofluorescence technique

The detection of ANXA1 and cell markers in the MDSC were performed in skin biopsies of patients by immunofluorescence technique, according to Silva and collaborators [25]. For ANXA1 detection, the antibody rabbit anti-ANXA1 [Invitrogen, USA; 1:200 in PBS/bovine serum albumin (BSA) at 1%] was used.

For identification of M-MDSC, it was used a monoclonal mouse IgG anti-CD14 (SANTA CRUZ biotechnology, 1:100 in 1% BSA), rat anti-MHCII (sc-59318; Santa Cruz Biotechnology Inc., Santa Cruz, CA 1:100), goat anti-CD11b (1:50 in 1% BSA).

For identification of G-MDSC, it was used monoclonal mouse IgM anti-CD15 (SANTA CRUZ biotechnology, 1:100 in 1% BSA), mouse anti-MHCII (sc-59318; Santa Cruz Biotechnology Inc., Santa Cruz, CA 1:100), goat anti-CD11b (Abcam 1:50 in 1% BSA).

As secondary antibodies, it was used the following: goat anti-rabbit IgG conjugated to Alexafluor 488 fluorochrome (Invitrogen, USA, 1:200 in 1% BSA), goat anti-mouse IgG conjugated to Alexafluor 555 (Invitrogen, USA, 1:200 in 1% BSA), goat anti-mouse IgM conjugated to Alexafluor 555 (Invitrogen, USA, 1:125 in 1% BSA), goat anti-mouse IgG conjugated to Alexafluor 633 (Invitrogen, USA, 1:50 in 1% BSA), and donkey anti-goat IgG conjugated to Alexafluor 350 (Invitrogen, USA, 1:25 in 1% BSA). The secondary antibodies were incubated for 1 h at room temperature and in the darkroom.

Twenty fields were analysed in each patient dermis for MDSC analysis. Up to three cells per field were considered a low number. More than four cells per field were considered a high number. After identification, two blinded observers quantified the cytoplasmic content of ANXA1 using the Axiovision software (Carl Zeiss, GR) by optical density average. The expression quantification was measured according to the light spectrum, ranging arbitrarily from 0 to 255 (arbitrary units—a.u.). ANXA1 values were expressed as mean ± standard error of the mean (SEM) in each MDSC.

### Statistical analysis

All data is provided in Additional file 1: Table S1. The sample size was calculated using Software IBM SPSS Statistics version 22, considering a 90% confidence interval and sample power > 80%. Population size was referred from data obtained from the State Health Secretary of Mato Grosso. Statistical analyzes were performed using the chi-squared test and Fischer’s exact test with a p-value less than 0.05 (< 0.05) for the estimated statistical associations.

The ANXA1 results obtained were statistically compared with the aid of the software GraphPad Prism 5 (La Jolla, CA, USA) through the analysis of variance (Oneway ANOVA) with Bonferroni post-test. p values less than 0.05 were considered statistically significant.

## Results

### Clinical and histopathological data

The patients were evaluated by clinical data and bacilloscopy, being classified as follows: 40 patients TT, 40 BB, 40 LL, 25 T1R and, 25 T2R. Men were the predominant sex (55.9%), and brown was the predominant skin color (54.1%). The majority of patients (56.5%) were 40–59 years old (Table 1).

**Table.1:** Sociodemographic profile of leprosy patients

Variables analyzed	Quantity	Percentage
Sex		
Male	95	55.9%
Female	75	44.1%
Skin color		
Black	41	24.1%
Brown	92	54.1%
Caucasian	37	21.8%
Age		
18–39	43	25.3%
40–59	96	56.5%
60–70	31	18.2%

### Analyzes of the bacilloscopy index (BI)

TT patients presented bacilloscopy index varied from 0 to 2 + , and 37.5% had 2 + . BB patients showed BI = 3 + to 4 + , and 90.0% had 3 + . LL patients showed BI = 5 + to 6 + , and 65.0% had 5 + . The patients T1R showed BI = 3 + to 4 + , and 64.0% had 3 + . The T2R had between BI = 4 + to 6 + , and 72.0% had 4 + (Table 2).

**Table.2:** Analysis of the bacilloscopic index found in the skin lesions of leprosy patients with tuberculoid, borderline, lepromatous, type 1 reaction (T1R) and type 2 reaction

Bacilloscopic index	TT	BB	LL	T1R	T2R
0	11	–	–	–	–
1 +	14	–	–	–	–
2 +	15	–	–	–	–
3 +	–	36	–	16	-
4 +	–	4	–	9	18
5 +	–	–	26	–	5
6 +	–	–	14	–	2
Total	40	40	40	25	25

### M-MDSC and G-MDSC in LL, T1R, and T2R lesion.

The presence of M-MDSC (CD14^+^CD11b^+^MHCII^±^) and G-MDSC (CD15^+^CD11b^+^MHCll^±^) in skin lesions of leprosy patients were evaluated. The majority of LL and T2R patients have a high number of M-MDSC and G-MDSC cells per field (66.6% and 50.0% in LL; 61.9% and 80.9% in T2R). T1R patients have a high number of M-MDSC (66.6%) in the skin lesion (Table 3).

**Table.3:** Quantity of MDSC in leprosy patients with borderline, lepromatous, type 1 reaction (T1R) and type 2 reaction

	Cell number		*p*
	Low	High	
M-MDSC			
BB	6	0	0.098
LL	12	28	
T1R	9	16	
T2R	8	17	
G-MDSC			
LL	15	25	0.002
T1R	19	6	
T2R	5	20	

Statistical analyzes were performed using the chi-squared test and Fischer’s exact test. LL, T1R and T2R patients showed a *p* = 0.098 for the M-MDSC high number. Also T2R patients showed a *p* = 0.002 for the G-MDSC high number

### Analyzes of ANXA1 expression in M-MDSC and G-MDSC

**Fig. 1:**
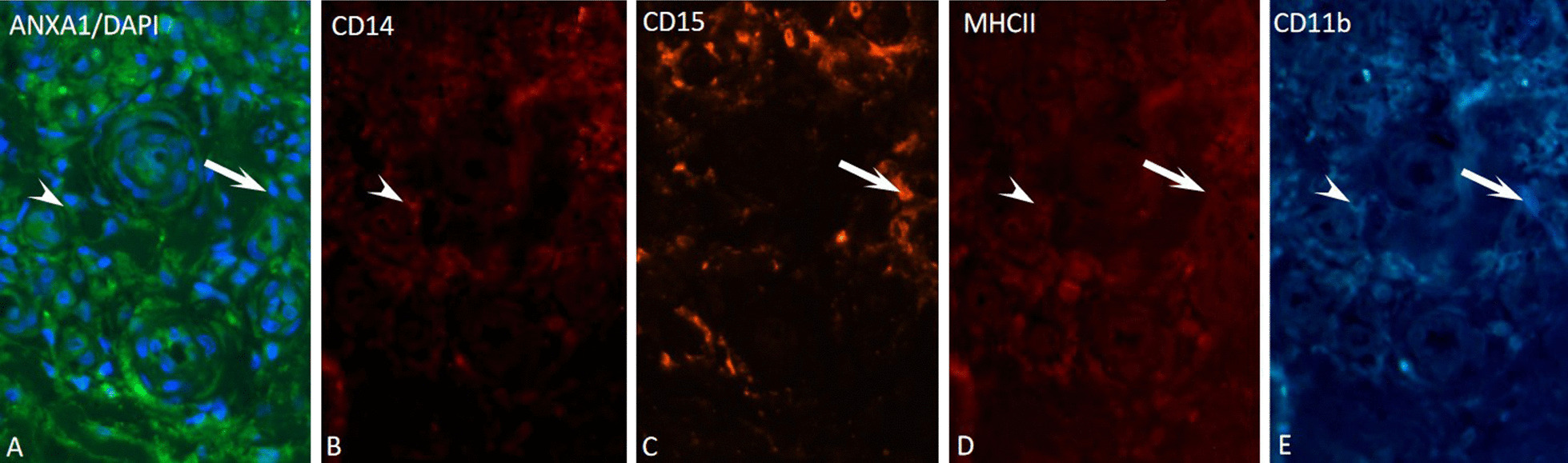
Analysis of immunofluorescence for annexin A1 in M-MDSC and G-MDSC in lepromatous leprosy patient’s skin lesion. M-MDSC cells (arrowhead) were immunostained for annexin A1 (**A**), CD14^+^ (**B**), MHC-II^±^ (**D**) and, CD11b^+^ (**E**). G-MDSC cells (arrow) were immunostained for annexin A1 (**A**), CD15^+^ (**C**), MHC-II^±^ (**D**) and, CD11b^+^ (**E**). Bar = 20 µm

The presence of ANXA1 was observed in all MDSC (Fig. 1). M-MDSC in the LL has higher levels of ANXA1 (125.5 ± 4.1 u.a) when compared to the reactive patients (Table 4). Also, the ANXA1 expression in T1R was the lowest when compared to T2R (Table 4).

**Table.4:** Analysis of annexin A1 expression in M-MDSC and G-MDSC at skin lesions of patients with leprosy

	M-MDSC	G-MDSC
LL	125.5 ± 4.1	110.8 ± 5.7
T1R	54.2 ± 4.3***	109.0 ± 4.6
T2R	91.8 ± 4.3*** ^###^	106.1 ± 3.9

Statistical analysis were verified by Oneway ANOVA with Bonferroni post-test. Annexin A1 expression in M-MDSC indicated a *p* value < 0.0001 (***) when compared with lepromatous leprosy patients. This protein in M-MDSC indicate a *p* < 0.0001 (^###^) when compared with T1R patients

The analysis of ANXA1 in G-MDSC (Fig. 1) showed that LL, T1R, and T2R have similar levels (ex: LL: 110.8 ± 5.7 u.a) (Table 4).

## Discussion

This study identified the presence of MDSCs in patients with leprosy and its expression of ANXA1 to establish a possible default association in leprosy reactions.

The epidemiological data showed that males were more affected than females. These data are consistent with the findings reported in the literature [26–28]. Several factors contribute to this scenario: lower health care dispensed, lifestyle factors, less concern with the self. Altogether, it may contribute to late diagnosis and, subsequently, disease dissemination.

In the present study a higher number of MDSCs were observed in LL and T2R compared to TR patients. The literature suggested that infectious diseases might inhibit the maturation of myeloid cells in the bone marrow, inducing migration to the inflammatory site, and differentiation as suppressor cells [29, 30]. The presence of these cells in patients LL alone can reduce the efficiency of the immunological system to fight against *M. leprae*. This data suggest that the fundamental role of MDSCs in the regulation of inflammatory reaction. Some studies say M-MDSC induces the proliferation of macrophages M2-like in hypoxic tumour areas [15] and contributes to the extracellular matrix remodelling [29]. Also, M-MDSC produces reactive oxygen species, which disrupts the T-cell function by modifying its TCR-ζ chain [17]. Regarding the G-MDSC, it mediates an immunosuppressive pattern through STAT6 signalling and expression of ARG-1 and TGF-β [10]. Also, G-MDSC induces the activation of Treg cells through IFN-γ and IL-10 [14].

Finally, to analyze a possible mechanism of action of MDSCs in patients with leprosy, the ANXA1 expression was analyzed. Previous studies have already demonstrated the ANXA1 expression in leukocytes of leprosy patients [31, 32]. However, this is the first study that highlights the presence of this protein in MDSCs. The data showed that the ANXA1 levels found in M-MDSCs were higher in LL patients when compared to the T1R and T2R, while similar levels were observed in G-MDSC at all patients. The literature shows that the ANXA1 is an endogenous regulatory protein expressed at high concentrations in granulocytes, particularly neutrophils [33–36]. The lower levels of ANXA1 in M-MDSCs of T1R and T2R might be due to drug treatment. This result can be an important limitation of this work. In particular, literature shows that, after 24 h of glucocorticoids treatment, ANXA1 expression reduces in macrophages [34]. Therefore, the high levels of this protein, observed in G-MDSC, might be linked to the type of cell lineage. It is well known that the ANXA1 has a modulatory role in the innate and adaptive immune response. Studies with ANXA1 knockout animals show acute and systemic inflammation exacerbation by pro-inflammatory TNF-α, IL-1β, and IL-6 release [18, 33, 34]. Also, ANXA1 is involved in the induction of IL-10 production [18, 37, 38]. This cytokine is produced by MDSCs and is an essential molecule in immune system regulation. This data suggest that the high ANXA1 expression in MDSCs at LL and T2R patients may be related to the regulation of infectious response, reducing the effectiveness of T cells action, and establishing an anergic response, leading patient susceptible to *M. leprae*.

The clinical evolution of leprosy is directly involved with the participation of pro-inflammatory mediators, which direct the immune response to a cellular or humoral profile. Some of these have their role well elucidated in the literature, However, there are gaps related to issues of resistance or susceptibility to individuals exposed to the bacillus. It was demonstrated the presence and importance of MDSC that can influence the host response against leprosy. Many issues and challenges are still open for the research of MDSCs and their role in leprosy.

## Conclusion

A high number of M-MDSC and G-MDSC was present in the skin lesions of patients with LL and T2R, whereas, in T1R patients, a high number of M-MDSC was observed. These data indicated a different mechanism of recruitment of those cells dependent on immunological outcomes.

LL patients expressed more ANXA1 in M-MDSCs than the patients T1R and T2R, possibly indicating the involvement of this protein in the anergic immune status of LL patients.

## Supplementary Information

**Additional file 1.** Supplementary table 1.

## Data Availability

All data generated or analysed during this study are included in this published article and in the Additional file 1: Table S1. Further datasets used and/or analysed during the current study are available from the corresponding author on reasonable request.

## Abbreviations

a.u: Arbitrary units
ANXA1: Annexin-A1
Arg: Arginine
BAAR: Acid-alcohol-resistant bacilli
BB: Borderline
BSA: Bovine serum albumin
ENL: Erythema nodosum leprosum
G-MDSC: Granulocyte myeloid-derived suppressor cell
IFN-γ: Interferon gamma
IL: Interleukin
iNOS: Inducible NO synthase
LL: Lepromatous
MDSC: Myeloid-derived suppressor cell
M-MDSC: Monocytic myeloid-derived suppressor cell
NO: Nitric oxide
PBS: Phosphate buffer saline
ROS: Reactive oxygen species
SEM: Standard error of the mean
T1R: Type 1 reaction
T2R: Type 2 reaction
TGF-β: Transforming growth factor beta
TT: Tuberculoid
UHJM: University Hospital Júlio Müller
