# Supplementary material for: Analysis of the myeloid-derived suppressor cells and annexin A1 in multibacillary leprosy and reactional episodes
Source: BMC Infect Dis. 2021 Oct 9;21:1050. doi: 10.1186/s12879-021-06744-x (PMC8502368; doi:10.1186/s12879-021-06744-x)
Supplement: Supplementary file 1 — Additional file 1. Supplementary table 1. [file 12879_2021_6744_MOESM1_ESM.docx]

**Supplementary Table 1. Data of leprosy patients: skin colour, sex, age, bacilloscopic index, BCG scar and number of M-MDSC and G-MDSC.**

|  | Clinical | Skin colour | Sex | Age | BI | M-MDSC | G-MDSC |
| --- | --- | --- | --- | --- | --- | --- | --- |
| 1 | TT | Brown | F | 18 to 29 | 1 | - | - |
| 2 |  | Brown | F |  | 2 | - | - |
| 3 |  | Caucasian | M |  | 1 | - | - |
| 4 |  | Caucasian | F |  | 0 | - | - |
| 5 |  | Brown | F | 30 to 49 | 2 | - | - |
| 6 |  | Brown | M |  | 1 | - | - |
| 7 |  | Brown | F |  | 1 | - | - |
| 8 |  | Caucasian | M |  | 0 | - | - |
| 9 |  | Brown | F |  | 1 | - | - |
| 10 |  | Brown | F |  | 0 | - | - |
| 11 |  | Caucasian | F |  | 0 | - | - |
| 12 |  | Brown | F |  | 2 | - | - |
| 13 |  | Brown | M |  | 2 | - | - |
| 14 |  | Caucasian | F |  | 1 | - | - |
| 15 |  | Brown | F |  | 2 | - | - |
| 16 |  | Brown | F |  | 0 | - | - |
| 17 |  | Caucasian | F |  | 1 | - | - |
| 18 |  | Brown | M |  | 1 | - | - |
| 19 |  | Black | M |  | 2 | - | - |
| 20 |  | Black | F |  | 1 | - | - |
| 21 |  | Brown | M | 50 to 66 | 1 | - | - |
| 22 |  | Black | F |  | 2 | - | - |
| 23 |  | Brown | F |  | 0 | - | - |
| 24 |  | Caucasian | F |  | 2 | - | - |
| 25 |  | Caucasian | F |  | 1 | - | - |
| 26 |  | Caucasian | F |  | 1 | - | - |
| 27 |  | Brown | F |  | 0 | - | - |
| 28 |  | Brown | F |  | 2 | - | - |
| 29 |  | Brown | M |  | 1 | - | - |
| 30 |  | Brown | F |  | 2 | - | - |
| 31 |  | Brown | M |  | 2 | - | - |
| 32 |  | Brown | F |  | 2 | - | - |
| 33 |  | Caucasian | F |  | 0 | - | - |
| 34 |  | Black | F |  | 2 | - | - |
| 35 |  | Caucasian | M |  | 1 | - | - |
| 36 |  | Brown | M |  | 0 | - | - |
| 37 |  | Brown | M |  | 2 | - | - |
| 38 |  | Caucasian | F |  | 0 | - | - |
| 39 |  | Black | M |  | 2 | - | - |
| 40 |  | Caucasian | F |  | 0 | - | - |
| 1 | BB | Brown | F | 19 to 29 | 3 | - | - |
| 2 |  | Brown | M |  | 3 | 1 | - |
| 3 |  | Brown | M |  | 3 | - | - |
| 4 |  | Brown | M |  | 3 | - | - |
| 5 |  | Black | F | 30 to 49 | 3 | - | - |
| 6 |  | Brown | M |  | 4 | 1 | - |
| 7 |  | Black | F |  | 3 | - | - |
| 8 |  | Caucasian | F |  | 3 | - | - |
| 9 |  | Black | M |  | 3 | - | - |
| 10 |  | Black | F |  | 3 | - | - |
| 11 |  | Brown | M |  | 3 | - | - |
| 12 |  | Brown | F |  | 3 | - | - |
| 13 |  | Black | F |  | 3 | - | - |
| 14 |  | Caucasian | F |  | 3 | - | - |
| 15 |  | Brown | F |  | 4 | 2 | - |
| 16 |  | Brown | M |  | 3 | - | - |
| 17 |  | Black | M |  | 3 | - | - |
| 18 |  | Black | M |  | 3 | - | - |
| 19 |  | Brown | M |  | 3 | - | - |
| 20 |  | Brown | F |  | 3 | - | - |
| 21 |  | Black | M |  | 3 | - | - |
| 22 |  | Brown | F |  | 4 | 2 | - |
| 23 |  | Caucasian | F |  | 3 | - | - |
| 24 |  | Brown | M |  | 3 | - | - |
| 25 |  | Caucasian | M |  | 3 | - | - |
| 26 |  | Black | M | 50 to 67 | 4 | - | - |
| 27 |  | Black | M |  | 3 | - | - |
| 28 |  | Brown | F |  | 3 | - | - |
| 29 |  | Black | F |  | 3 | - | - |
| 30 |  | Brown | M |  | 3 | - | - |
| 31 |  | Brown | F |  | 3 | - | - |
| 32 |  | Black | F |  | 3 | - | - |
| 33 |  | Brown | M |  | 3 | 1 | - |
| 34 |  | Brown | F |  | 3 | - | - |
| 35 |  | Brown | M |  | 3 | - | - |
| 36 |  | Black | M |  | 3 | - | - |
| 37 |  | Brown | M |  | 3 | - | - |
| 38 |  | Brown | F |  | 3 | - | - |
| 39 |  | Caucasian | M |  | 3 | - | - |
| 40 |  | Black | M |  | 3 | 1 | - |
| 1 | LL | Black | M | 18 to 29 | 5 | 6 | 5 |
| 2 |  | Brown | M |  | 5 | 2 | 2 |
| 3 |  | Black | M |  | 6 | 10 | 7 |
| 4 |  | Black | M |  | 6 | 7 | 6 |
| 5 |  | Brown | M |  | 6 | 5 | 5 |
| 6 |  | Black | F | 30 to 49 | 5 | 6 | 5 |
| 7 |  | Brown | F |  | 5 | 3 | 3 |
| 8 |  | Brown | M |  | 5 | 4 | 6 |
| 9 |  | Brown | F |  | 6 | 8 | 8 |
| 10 |  | Black | M |  | 6 | 7 | 6 |
| 11 |  | Caucasian | F |  | 5 | 8 | 7 |
| 12 |  | Brown | M |  | 5 | 6 | 7 |
| 13 |  | Brown | M |  | 5 | 6 | 6 |
| 14 |  | Black | M |  | 6 | 8 | 6 |
| 15 |  | Brown | M |  | 6 | 2 | 1 |
| 16 |  | Brown | F |  | 5 | 7 | 5 |
| 17 |  | Brown | F |  | 5 | 9 | 7 |
| 18 |  | Brown | M |  | 6 | 2 | 2 |
| 19 |  | Caucasian | M |  | 6 | 4 | 3 |
| 20 |  | Caucasian | F |  | 6 | 2 | 2 |
| 21 |  | Black | M |  | 6 | 2 | 2 |
| 22 |  | Black | M | 50 to 66 | 5 | 7 | 4 |
| 23 |  | Black | M |  | 5 | 5 | 7 |
| 24 |  | Black | M |  | 5 | 6 | 4 |
| 25 |  | Caucasian | M |  | 5 | 6 | 7 |
| 26 |  | Caucasian | M |  | 5 | 9 | 7 |
| 27 |  | Caucasian | M |  | 5 | 2 | 1 |
| 28 |  | Brown | F |  | 5 | 3 | 1 |
| 29 |  | Brown | M |  | 5 | 2 | 3 |
| 30 |  | Brown | F |  | 5 | 2 | 2 |
| 31 |  | Brown | F |  | 5 | 4 | 2 |
| 32 |  | Black | F |  | 5 | 4 | 5 |
| 33 |  | Black | F |  | 5 | 3 | 3 |
| 34 |  | Brown | F |  | 5 | 5 | 4 |
| 35 |  | Black | M |  | 6 | 5 | 4 |
| 36 |  | Brown | M |  | 5 | 3 | 3 |
| 37 |  | Black | M |  | 5 | 6 | 4 |
| 38 |  | Brown | M |  | 6 | 4 | 3 |
| 39 |  | Black | M |  | 5 | 4 | 4 |
| 40 |  | Brown | M |  | 6 | 6 | 6 |
| 1 | T1R | Brown | F | 18 to 29 | 3 | 7 | 1 |
| 2 |  | Caucasian | M | 18 | 4 | 6 | 4 |
| 3 |  | Brown | F | 30 to 49 | 3 | 1 | 2 |
| 4 |  | Caucasian | M |  | 3 | 8 | 1 |
| 5 |  | Caucasian | M |  | 3 | 6 | 2 |
| 6 |  | Caucasian | F |  | 3 | 8 | 2 |
| 7 |  | Caucasian | F |  | 4 | 2 | 1 |
| 8 |  | Black | M |  | 3 | 3 | 1 |
| 9 |  | Brown | M |  | 4 | 4 | 5 |
| 10 |  | Brown | M |  | 3 | 7 | 1 |
| 11 |  | Brown | M | 50 to 70 | 4 | 8 | 4 |
| 12 |  | Brown | M |  | 3 | 6 | 4 |
| 13 |  | Brown | F |  | 4 | 6 | 5 |
| 14 |  | Brown | M |  | 4 | 7 | 2 |
| 15 |  | Black | M |  | 3 | 3 | 2 |
| 16 |  | Brown | M |  | 3 | 7 | 1 |
| 17 |  | Caucasian | M |  | 3 | 1 | 1 |
| 18 |  | Brown | F |  | 4 | 2 | 2 |
| 19 |  | Black | M |  | 3 | 3 | 2 |
| 20 |  | Brown | M |  | 3 | 8 | 2 |
| 21 |  | Brown | F |  | 3 | 4 | 1 |
| 22 |  | Brown | F |  | 4 | 2 | 1 |
| 23 |  | Caucasian | M |  | 4 | 4 | 5 |
| 24 |  | Caucasian | M |  | 3 | 2 | 2 |
| 25 |  | Black | M |  | 3 | 7 | 1 |
| 1 | T2R | Black | M | 18 to 29 | 4 | 7 | 7 |
| 2 |  | Brown | M |  | 4 | 9 | 10 |
| 3 |  | Caucasian | M | 30 to 49 | 4 | 10 | 9 |
| 4 |  | Brown | M |  | 4 | 8 | 7 |
| 5 |  | Brown | F |  | 4 | 10 | 9 |
| 6 |  | Black | M |  | 4 | 3 | 5 |
| 7 |  | Brown | F |  | 4 | 8 | 7 |
| 8 |  | Brown | F |  | 4 | 8 | 6 |
| 9 |  | Brown | M |  | 4 | 7 | 5 |
| 10 |  | Brown | M |  | 4 | 4 | 3 |
| 11 |  | Brown | F |  | 5 | 3 | 2 |
| 12 |  | Brown | F |  | 5 | 7 | 5 |
| 13 |  | Caucasian | M |  | 4 | 5 | 5 |
| 14 |  | Brown | F |  | 4 | 3 | 5 |
| 15 |  | Brown | M |  | 4 | 7 | 5 |
| 16 |  | Brown | F | 50 to 69 | 5 | 8 | 6 |
| 17 |  | Brown | M |  | 4 | 3 | 7 |
| 18 |  | Caucasian | M |  | 5 | 2 | 5 |
| 19 |  | Brown | M |  | 4 | 7 | 7 |
| 20 |  | Brown | M |  | 5 | 10 | 8 |
| 21 |  | Brown | F |  | 4 | 6 | 5 |
| 22 |  | Caucasian | M |  | 6 | 6 | 5 |
| 23 |  | Brown | F |  | 6 | 3 | 2 |
| 24 |  | Caucasian | M |  | 4 | 3 | 2 |
| 25 |  | Black | M |  | 4 | 2 | 1 |

F = feminine, M = masculine, N = no, Y = yes, BI = bacilloscopic index.
